# Supplementary material for: Route to high-energy density polymeric nitrogen t-N via He−N compounds
Source: Nat Commun. 2018 Feb 19;9:722. doi: 10.1038/s41467-018-03200-4 (PMC5818478; doi:10.1038/s41467-018-03200-4)
Supplement: Supplementary file 1 — Supplementary Information [file 41467_2018_3200_MOESM1_ESM.doc]

**Route to High-Energy Density Polymeric Nitrogen, *t*-N, via He-N Compounds**

Li et al.

Supplementary Table 1 Calculated lattice parameters for various HeN_4_ phases at selected pressures. Atomic positions are in fractional coordinates. Lattice parameters are in units of Å.

| Structure | Pressure (GPa) | Lattice parameters | Atomic Position |
| --- | --- | --- | --- |
| *C*2/*c* | 25 | *a* = 9.1249  *b* = 5.6497  *c* = 6.1071  *β* = 114.87° | He (4*e*) (0, -0.758, 0.75)  N (8*f*) (0.33, -0.626, 0.366)  N (8*f*) (-0.198, -0.443, -0.33) |
| *P*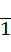 | 80 | *a* = 2.7717  *b* = 3.7601  *c* = 5.5805  *α* = 84.8°  *β* = 98.52°  *γ* = 98.15° | He (2*i*) (-0.779, -0.12, -0.999)  N1 (2*i*) (-0.383,-0.341,-0.47)  N2 (2*i*) (0.167, -0.808, -0.347)  N3 (2*i*) (0.567, -0.293, -0.249)  N4 (2*i*) (0.285,-0.577, -0.183) |
| *I*4_1_/*a* | 100 | *a* = 4.8509  *c* = 4.1832 | He (4*b*) (0.5, 0, 0.25)  N (16*f*) (-0.769, 0.355, -0.605) |

Supplementary Table 2 Calculated lattice parameters for *t*-N_2_ phase at ambient pressure. Atomic positions are in fractional coordinates. Lattice parameters are in units of Å.

| Structure | Lattice parameters | Atomic Position |
| --- | --- | --- |
| *I*4_1_/*a* | *a* = 5.461  *c* = 3.8873 | N (16*f*) (0.706, 0.867, 0.939) |

Supplementary Table 3 Calculated Mulliken Population of HeN_4_ at selected pressures. While calculations for charge transfer for such solids are dependent to some extent on the basis set adopted, these values allow comparisons between structures and demonstrate qualitatively the weak charge transfer between He and N.

| Structure | P (GPa) | Atom | s (*e*) | p (*e*) | Charge (*e*) |
| --- | --- | --- | --- | --- | --- |
| *C*2/*c* | 25 | He  N  N | 2  1.69  1.7 | 3.31  3.3 | 0  0  0 |
| *P*$\bar{1}$ | 80 | He  N1  N2  N3  N4 | 2  1.27  1.58  1.56  1.59 | 3.56  3.54  3.48  3.45 | 0  0.17  -0.11  -0.03  -0.03 |
| *I*4_1_/*a* | 100 | He  N | 2  1.22 | 3.78 | 0  0 |

Supplementary Table 4 Calculated elastic constant *C_ij_* (GPa), bulk modulus *B* (GPa) and shear modulus *G* (GPa) of *t*-N_2_ at ambient pressure.

|  | *C*_11_ | *C*_33_ | *C*_44_ | *C*_66_ | *C*_12_ | *C*_13_ | *B*_0_ | *G* |
| --- | --- | --- | --- | --- | --- | --- | --- | --- |
|  | 293 | 307 | 236 | 162 | 63 | 113 | 163 | 167 |

Supplementary Table 5 Calculated Bond Population of HeN_4_ and *t*-N at selected pressures.

| Structure | P (GPa) | Bond | Length (Å) | Population |
| --- | --- | --- | --- | --- |
| *C*2/*c* | 25 | N-N | 1.107 | 1.52 |
| *P*$\bar{1}$ | 80 | N-N  N-N  N-N  N-N | 1.292  1.293  1.301  1.320 | 0.84  0.76  0.72  0.67 |
| *I*4_1_/*a* | 100 | N-N  N-N | 1.278  1.426 | 0.87  0.51 |
| *t*-N | 0 | N-N  N-N | 1.368  1.614 | 0.69  0.35 |

Supplementary Table 6 Calculated interaction energy (Δ*H*_int_) of the three phases of HeN_4_ at different pressure with the equation Δ*H*_int_=*H*_HeN4_-(*H*_N_-*H*_He_).

| Structure | P (GPa) | Δ*H*_int_ (eV/atom) |
| --- | --- | --- |
| *C*2/*c* | 25 | -0.033 |
| *P*$\bar{1}$ | 70 | -0.012 |
| *I*4_1_/*a* | 100 | -0.07 |


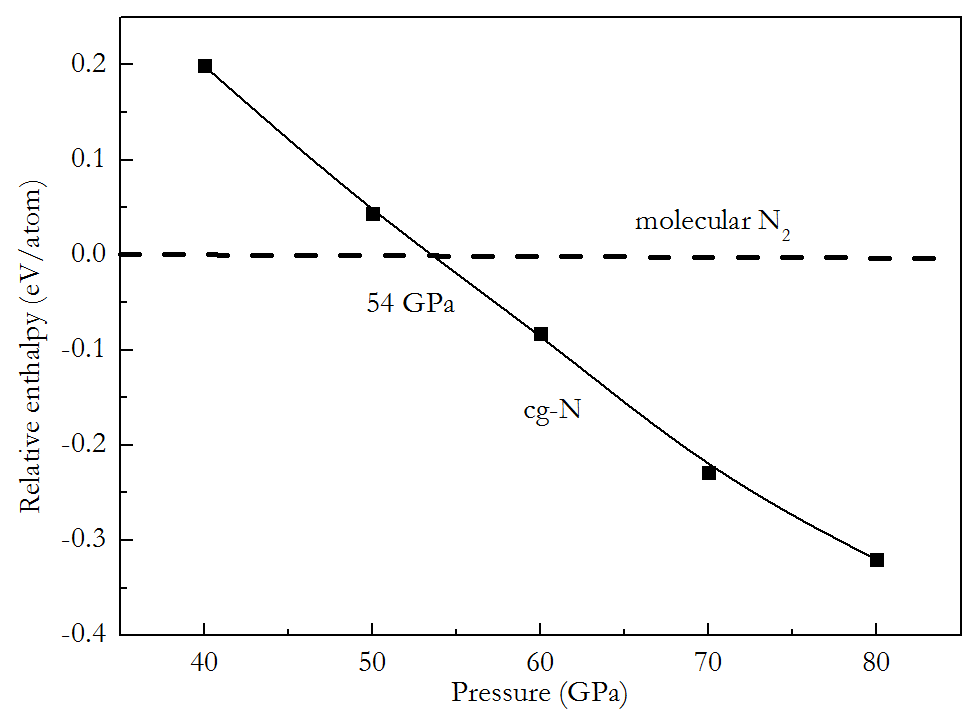


Supplementary Figure 1 Calculated relative enthalpy of *cg*-N with respect to molecular *ε*-N_2_ as a function of pressure, revealing a phase transition at 54 GPa with a sharp decrease of its enthalpy.


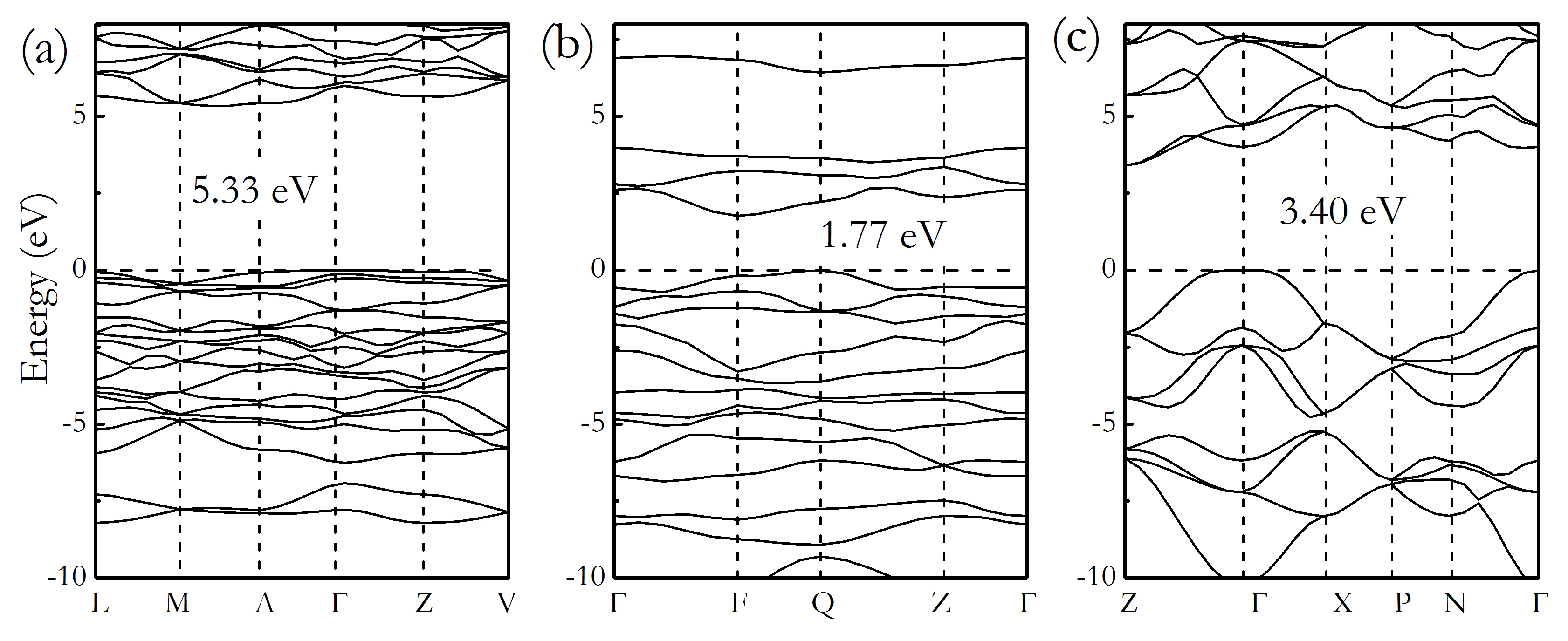


Supplementary Figure 2 Electronic band structures of HeN_4_. (a) the C2/c structure at 25 GPa, (b) the *P*
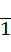
 structure at 80 GPa and (c) the I4_1_/a structure at 100 GPa.


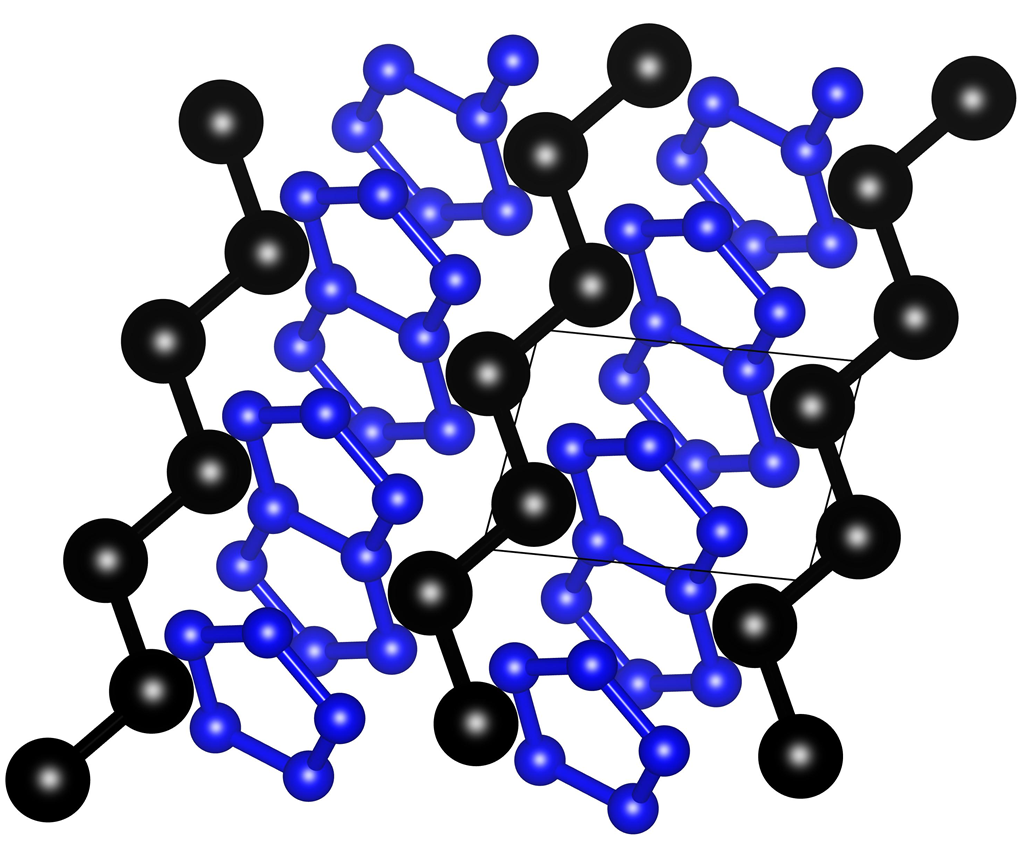


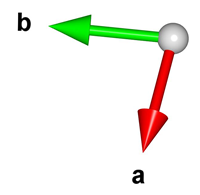


Supplementary Figure 3 Schematic of the *P*
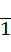
 structure of HeN_4_ showing the He-He zig-zag chains in layers parallel to (001). Small blue and large black spheres represent N and He atoms, respectively.


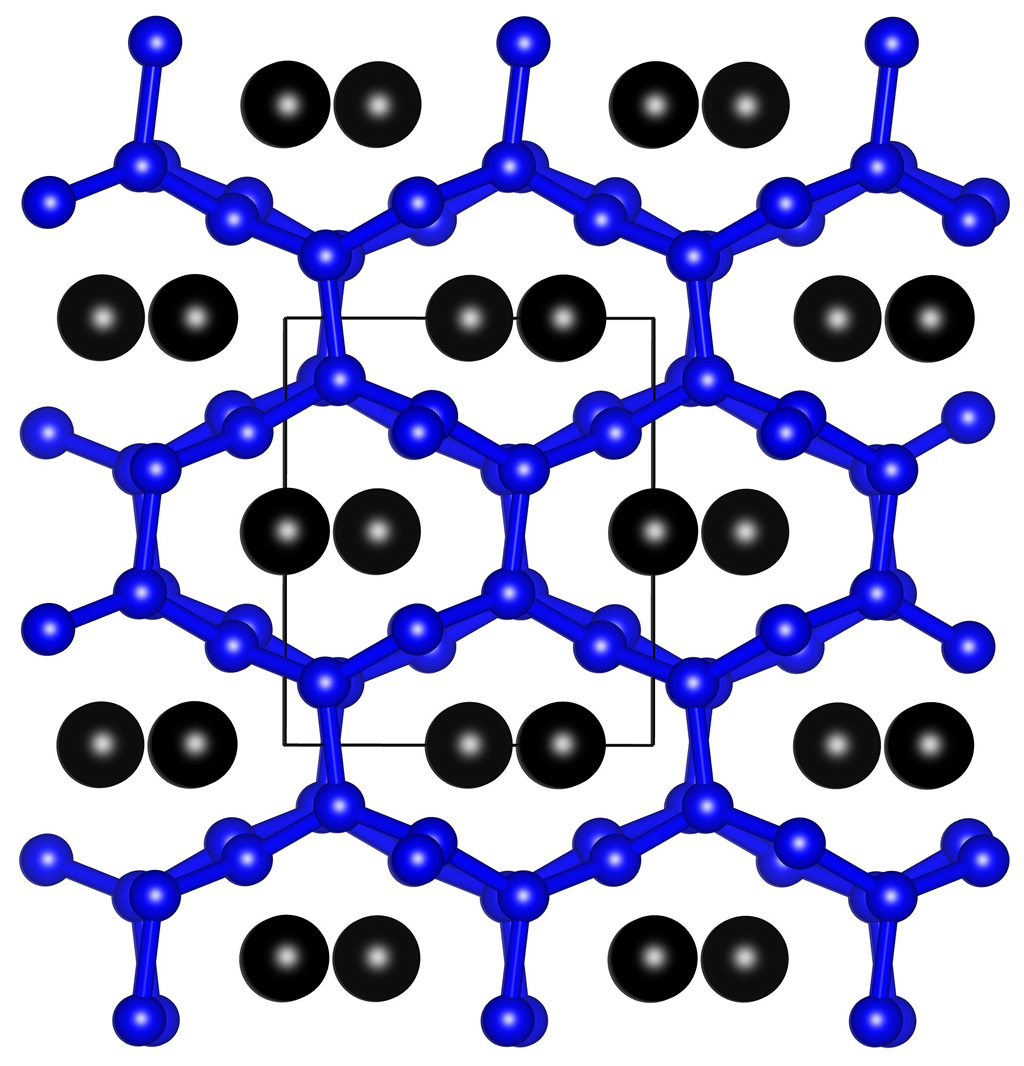


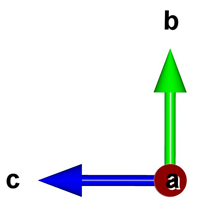


Supplementary Figure 4 Schematic of the *I*4_1_/*a* structure of HeN_4_ along *a*-axis showing the N_10_-rings. Small blue and large black spheres represent N and He atoms, respectively.


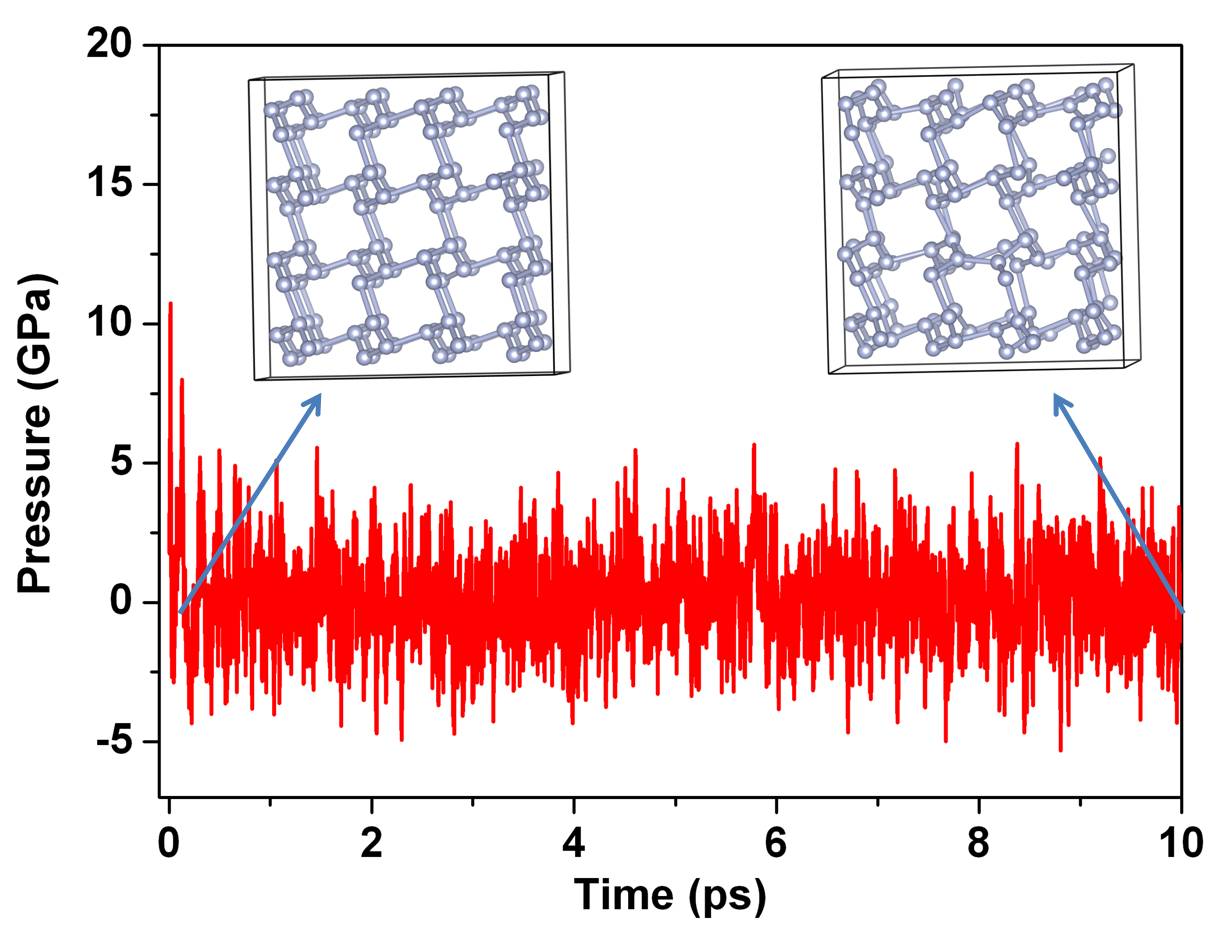


Supplementary Figure 5 Pressure as a function of time in NVT-MD simulation at 1000K. The *t*-N *I*4_1_/*a* structure did not collapse after 10 ps.
